# Neuropathic Pain in Aged People: An Unresolved Issue Open to Novel Drug Approaches, Focusing on Painful Diabetic Neuropathy

**DOI:** 10.2174/1570159X21666230807103642

**Published:** 2023-08-07

**Authors:** Nicoletta Marchesi, Foroogh Fahmideh, Alessia Pascale, Massimo Allegri, Stefano Govoni

**Affiliations:** 1 Department of Drug Sciences, Section of Pharmacology, University of Pavia, Pavia, Italy;; 2 Ensemble Hospitalier de la Cote - Centre Lemanique d’antalgie et Neuromodulation, Morges, Switzerland

**Keywords:** Older patients, neuropathic pain, diabetic neuropathy, dietary supplements, targeted therapy, pain management

## Abstract

A majority of older patients suffer from neuropathic pain (NP) that significantly alters their daily activities and imposes a significant burden on health care. Multiple comorbidities and the risk of polypharmacy in the elderly make it challenging to determine the appropriate drug, dosage, and maintenance of therapy. Age-dependent processes play a contributing role in neuropathy given that diabetic neuropathy (DN) is the most common form of neuropathy. This narrative review is mainly focused on the drug treatment approach for neuropathy-associated pain in aged people including both drugs and dietary supplements, considering the latter as add-on mechanism-based treatments to increase the effectiveness of usual treatments by implementing their activity or activating other analgesic pathways. On one hand, the limited clinical studies assessing the effectiveness and the adverse effects of existing pain management options in this age segment of the population (> 65), on the other hand, the expanding global demographics of the elderly contribute to building up an unresolved pain management problem that needs the attention of healthcare providers, researchers, and health authorities as well as the expansion of the current therapeutic options.

## 1 INTRODUCTION

From a conceptual point of view, the peculiar problem of aged people suffering from neuropathy-associated pain raises several questions which do not have yet ad hoc reference guidelines. Even in the systematic review and meta-analysis by Finnerup *et al.* (2015) on pharmacotherapy for neuropathic pain in adults, some indications for over 65 years old patients are reported [1]. Generally, the treatment of older patients affected by painful syndromes should privilege treatments that do not or scarcely influence motor and cognitive responses a point that needs careful and specific studies. In a 2019 meta-analysis, Allegri *et al.* reported that in non-cancer patients long-term opioids had relatively minor effects on cognitive function, in particular affecting attention [2]. On the other hand, the association between opioids and centrally-acting molecules (as some antiepileptics used in pain therapy) further compromised this parameter. Such an additive effect was not observed with non-centrally-acting substances. However, the screened studies did not adequately include older patients. Indeed, this aspect needs further studies purposely conducted on them. Similar studies targeting older patients with various pain syndromes could be used to generate strategies to refine pain treatments in this age group.

**Fig. (1):**
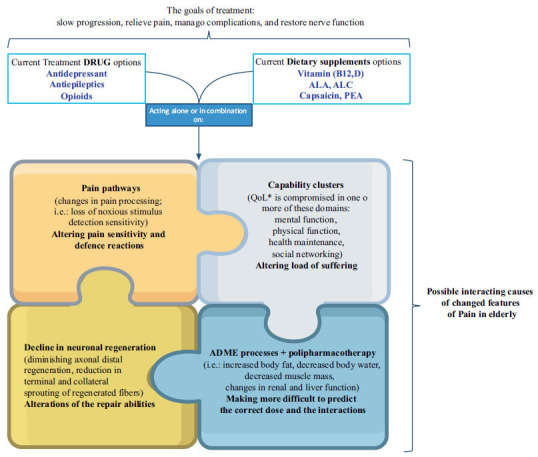
Neuropathic pain treatment in older patients: the interacting players (and confounders). **Abbreviations**: *QoL = Quality of life, ALC = Acetyl-L-carnitine, ALA = Alpha-lipoic acid, PEA = Palmitoylethanolamide.

Moreover, guidelines should be designed underscoring the importance of the use of drugs for pain treatment in older patients that do not have an excessive sedative load and also considering the importance of comorbidities and polypharmacotherapy, with attention to the international criteria designed to optimize the prescription in the older patients (as summarized from a general point of view in Fig. **1**). Finally, special care should be given to cognitively impaired and demented patients which may be more sensitive to the effects of the treatment on cognitive functioning (see for example Tsai *et al.*, 2021, 2022) requiring a balance between analgesia use and non-pharmacological pain management in dementia care [3, 4].

Within this context, neuropathic pain (NP) refers to a category of chronic pain conditions that are caused by disease or lesion of the somatosensory nervous system including peripheral fibres (Aβ, Aδ, and C fibres) and central neurons [5, 6]. NP is a common yet underestimated clinical problem in older patients. Researchers and clinicians dealing with pain must take extra care with older people since their prognosis is often less favourable, and they gradually lose their independency and suffer from a high number of comorbidities [7]. It is estimated that NP prevalence in the elderly lies in the 11-35% range [7-9], a value higher than in the general population presenting prevalence ranges between 7-10% [10]. Regardless of age, NP may be classified as peripheral, central, or mixed, depending on the location of the lesion or disease [6]. Pain caused by strokes, spinal cord injuries, spinal infarctions, multiple sclerosis, and Parkinson's disease is central NP. Peripheral NP encompasses conditions such as painful diabetic neuropathy, postherpetic neuralgia, nerve entrapment syndromes, and neurological conditions due to genetic, metabolic, and toxic factors including some antitumoral and cancer chemotherapy treatments like cisplatin as well as immune-mediated neuropathies. Alternatively, spinal stenosis, fibromyalgia (defined as nociplastic pain), cancer pain as well as postherpetic neuralgia may be caused by both central and peripheral mechanisms [6, 11-13]. Age intervenes as an independent risk factor for neuropathy, suggesting that age-dependent processes specifically play a contributing role [14]. Given that diabetic neuropathy (DN) is the most common form of neuropathy worldwide and advancing age is a strong risk factor for DN, which is the most prevalent chronic complication of diabetes [15, 16], this narrative review mostly is referred to this subtype of neuropathy-associated pain.

There is a positive correlation between age and prevalence (50%) of DN [17, 18] contributing to poor quality of life, inability to perform daily activities, impaired mobility, depression, insomnia, anorexia, and in general, exacerbating existing co-morbidities [19, 20]. Therefore, this narrative review focuses on both drugs and dietary supplements as approaches to treating or lessening neuropathy-associated pain with a particular focus on DN in aged people.

## 2 DIABETIC NEUROPATHIC PAIN PERCEPTION IN OLDER ADULTS: EFFECTS OF AGING ON ANATOMICAL, PHYSIOLOGICAL, AND BIOLOGICAL CHANGES

It should be highlighted that as people age, their peripheral and autonomic nervous systems undergo physiological changes which make the process of detecting subclinical neuropathy more challenging. Additionally, specific symptoms of DN may be more difficult to recognize because of many associated age-related diseases (such as neurological, rheumatic, or vascular disorders) commonly encountered in older patients [14, 21]. To detect early stages of diabetic neuropathic associated pain screening for symptoms and signs in clinical practice is critical. Clinical diagnosis is usually made based on worsening the pain at rest (typically at night or early in the morning), which further causes a reduction in sleep hours and poor sleep quality. Moreover, the pain can be associated with positive sensory alterations (the presence of burning, paresthesias, or allodynias) as well as negative ones (loss of sensitivity) [7, 22]. The nociceptive pain, if present, can mask neuropathic pain. Besides, aged people can often have difficulty reporting or communicating pain, even in the absence of cognitive impairment, therefore any changes in the behaviour should be taken into consideration [23, 24]. Although screening for more rare forms of diabetic neuropathy should be warranted, the most common and well-studied form of DN is distal symmetrical sensory polyneuropathy, which affects pain and temperature sense, and cardiovascular autonomic neuropathy which is associated with an increased risk of postural hypotension and coronary events [14, 16]. The former identifies a high risk for foot complications, such as ulcers and gangrene, often leading to amputation, while cardiac autonomic neuropathy identifies an increased risk of orthostatic hypotension and coronary events [14, 25].

As people age, their anatomical features change, such as the loss of neurons in the central nervous system, increased abnormal or degenerating fibers, and a slower conduction velocity. All this leads to an altered perception of neuropathic pain among aged patients. Furthermore, aging involves biological and physiological changes that can affect the sensation of neuropathic pain, for instance, altered endogenous inhibition and decreased function of neurotransmitters [7, 26]. Of interest, DN can be due to both the reasons, damage to nerve cells, and also to neuronal ischemia due to low neurovascular flow both caused by hyperglycaemia [27]. Indeed, aging affects the mechanism thought to be involved in DN. Oxidative stress, a consequence of hyperglycaemia, is indicated as a contributor to diabetic neuropathy and aging further exacerbates the condition [28]. Aging is associated with an increased level of subclinical systemic inflammatory markers, such as cytokines IL-6, TNF-α, and acute phase proteins such as CRP [29]. Moreover, among the causes of diabetic neuropathy, impaired blood flow, and endoneurial microvasculopathy in the form of blood vessel wall thickening or occlusion, play an important role [15, 30]. Within this context, with advancing age, blood vessel walls undergo structural and functional changes, which result in increased vascular stiffness and endothelial dysfunction. Such arterial stiffening is associated with decreased cerebral blood flow, increased cerebrovascular resistance the cerebrovascular conductance index, and subsequently hypoperfusion [31, 32].

Despite the advances in elucidating the impact of aging on the pathogenesis of DN, specific and strong features or molecular signatures that reveal this heterogeneous disease in the elderly remain lacking.

## 3 TREATING NEUROPATHIC PAIN IN OLDER PATIENTS

Treating neuropathic pain in older patients is challenging because aged people are more vulnerable than younger ones since they often suffer from multiple medical and nutritional problems. A multidisciplinary approach using multicomponent strategies is needed as the most efficacious and safest therapeutic option for this population. Prescribing physicians should start treating the geriatric population with the lowest possible, tolerable doses aimed at causing less adverse effects and improving the quality of life. This can limit treatment options with analgesic agents due to an increased risk of adverse effects and problems with complex drug interactions [33]. In addition, older persons may have substantial differences in the absorption and clearance of drugs [34]. Due to these factors, there is an increased risk of excess sedation, confusion, constipation, and urinary retention [35].

With aging changes in absorption, distribution across body compartments, metabolism, and excretion take place. Some changes are clinically relevant. The metabolism and excretion of many drugs decrease, requiring that doses of some drugs be decreased [36].

It is also acknowledged that older people take more medications and are more likely to have multiple medical problems, and this increases the risk of drug-drug and drug-disease interactions [34]. It is suggested that the rate of drug metabolism, which occurs mainly in the liver, is often reduced with advancing age. In drugs that are mainly excreted by the kidney, the rate of elimination is also generally reduced in the elderly. Therefore, the dose requirement for these types of drugs may be lower in many older patients [34]. Just as an example, this may be the case for drugs like pregabalin and gabapentin, as suggested in the general Beers criteria examining drug use in older adults or by Bates *et al.* while reviewing the management of neuropathic pain [37]. It goes without saying that due to the fragility and special needs of this population, more clinical and observational studies are needed. We lack proper studies to compare the efficacy, dose, and adverse effects of medications used for DN in the elderly, as shown in Table **1** [38-42].

In a paper published in 2017, Cruccu and Truini reported a synthesis of the treatment of neuropathic pain by examining clinical practice guidelines published by a number of international and regional professional associations. In general, the recommendations based on these and several other similar reviews come from randomized controlled trials from which it is usually difficult to extract information specific to older patients. The general recommendation is to commence treatment with calcium channel alpha-2-delta ligands like gabapentin/pregabalin or a tricyclic antidepressant (*e.g.*, amitriptyline or nortriptyline) or SNRIs such as duloxetine. The choice between a gabapentinoids and a tricyclic antidepressant is based on considerations of tolerability rather than quality or intensity of pain. The second-line treatments include tramadol and topical capsaicin or lidocaine. Reservations are expressed in relation to strong opioids or some antiepileptic drugs (*e.g.*, lamotrigine) which feature as third- or fourth-line choices [43].

Summarizing, pharmacological therapy for neuropathic pain in the elderly is often inadequate. Comorbidities can affect the proper management of chronic pain and its effects on elderly people. Some clinical conditions, such as chronic kidney disease or heart failure, require a careful evaluation of types, times, and dosages of pharmacological therapies. Older patients generally are affected by more than one illness and usually require chronic and multiple medications, which might interact with medication for persistent pain [44]. Accordingly, in aged patients, the management of neuropathic pain requires multi-disciplinary team assessment. Pain relief and the improvement of the consequences derived from persistent pain should be the main goals for those who approach this issue [45].

In general, the guidelines for neuropathic pain management suggest a substantial drug approach with no distinctions based on different ages. In these circumstances, pharmacologic therapies represent the first step in pain treatment. Also, in the case of painful diabetic neuropathy the treatment approach, provided a careful differential diagnosis, the guidelines rely on similar grounds and drugs, the goals of treatment being to slow progression, relieve pain, manage complications, and restore function. Many prescription medications are available for painful diabetic neuropathy, among which pregabalin is the recommended as first-line treatment in all five major international clinical guidelines for DN. In older patients, the occurrence of side effects especially cognitive dysfunction and peripheral oedema as well as drowsiness, dizziness, and swelling in the hands and feet raises concerns during long-term therapy [46, 47]. Recently, few studies have highlighted the role of oxcarbazepine, a second-generation antiepileptic, in painful DN [48]. In 2018 a prospective, randomized, single-blind, parallel-group study was done to compare pregabalin and oxcarbazepine monotherapy in patients of painful DN. This trial showed that both oxcarbazepine 600 mg/day and pregabalin 150 mg/day provided a significant degree of pain relief in peripheral diabetic neuropathy in older patients. Although patients on pregabalin therapy showed an earlier onset of therapeutic effect as compared to oxcarbazepine, the efficacy of the two drugs was found comparable during the 12 weeks of the follow-up period of the study [48]. In this study, the authors have chosen lower doses of oxcarbazepine (600 mg) and pregabalin (150 mg) monotherapy for older patients with painful DN, compared to the usual ones (600-1800 mg and 150-600 mg respectively) [47, 49], taking into consideration the fact that older patients showed an increased sensitivity to both drugs and a corresponding increase in the incidence of adverse effects [48].

Among other choices, some antidepressants ease nerve pain and might be effective in painful diabetic neuropathy and postherpetic neuralgia, even if the patient is not depressed. Tricyclic antidepressants may help with mild to moderate nerve pain. Drugs in this class include amitriptyline, nortriptyline, and desipramine. If a single drug approach does not give pain relief, considering that TCAs and gabapentinoids have different mechanisms, a combination of these medications may be useful in the management of neuropathic pain [50]. Considering the numerous bothersome adverse effects, such as cardiac conduction abnormalities, anticholinergic effects, and postural hypotension, which can contribute to falls and fractures, TCAs do not represent the first pharmacological choice among older people [50].

Serotonin and norepinephrine reuptake inhibitors (SNRIs) are another type of antidepressant that may help with nerve pain and have fewer side effects.

Both duloxetine and venlafaxine, are approved for the treatment of painful DN as alternative therapy when gabapentinoids are not efficacious. The American Diabetes Association (ADA) recommends duloxetine as a first-line treatment. Possible side effects include nausea, sleepiness, dizziness, decreased appetite, and constipation. Duloxetine should be taken at a starting oral dosage of 60 mg per day given in the morning. Among older people, a daily dosage of 30 mg/d could also be effective. It can cause nausea, vomiting, dizziness, somnolence, constipation, and increased blood pressure. Duloxetine is also given in combination with gabapentinoids to decrease the threshold of the neuropathic component of cancer pain. Another drug that may be used is venlafaxine [51]. Venlafaxine is found to be effective not only in painful DN but also in all the other forms of painful polyneuropathies [52]. In the short-acting formulation, a dosage of 25 mg is given two or three times a day. Considering the long-acting venlafaxine, the starting dose is 37.5 mg or 75 mg once a day. The total daily dosage may be increased to 225 mg a day. Side effects, such as sweating, cardiac conduction abnormalities, and high blood pressure are seen during the titration to the target dose. If a single-drug treatment is not effective for pain relief, adding venlafaxine to gabapentin may be more effective in the treatment of painful diabetic neuropathy [53]. This class of drugs shows its effectiveness not only on neuropathic pain but also on depressed mood, which is a common consequence due to persistent pain and comorbidity.

Sometimes, an antidepressant may be combined with an anti-seizure drug. These drugs can also be used with common pain-relieving medications available without a prescription such as an acetaminophen or ibuprofen or a skin patch with lidocaine (a numbing substance). Within this context, it should be noted that in the 2015 systematic analysis by Finnerup *et al.* (2015) [1] formulations in patches (capsaicin and lidocaine) were considered advantageous in frail and older patients because of the topical application, in neuropathic pain with presumed local pain generator, even if problems of skin permeability in older patients have to be adequately addressed. Another drug that is administered locally is the botulinum toxin. This drug has been evaluated as third-line therapy for neuropathic pain [1] and recently revised with a positive conclusion on the efficacy [54]. In the case of older patients caution is recommended in the case of prolonged treatments to limit the risk of systemic effects after local delivery [55].

## 4 IS THERE A ROLE FOR FOOD SUPPLEMENTS/MEDICAL DEVICES?

Can dietary supplements help? This is an emblematic and equivocal question. In order to manage diabetes and prevent or slow its complications, such as DN, a healthy diet, and sugar balance is crucial; therefore, dietary supplements aimed to control dietary sugar intake and insulin resistance may play a role. Besides pharmacological interventions, dietary supplements aimed at reducing DN and DN-associated pain may help to reduce DN symptoms in older patients. Accordingly, this part of the review summarizes the latest data on possible relationships between DN and several nutritional supplements such as Vitamin B_12_, Vitamin D, Acetyl-L-carnitine (ALC), Alpha-lipoic acid (ALA), capsaicin and Palmitoylethanolamide (PEA) as detailed below. Note that some dietary supplements may interfere with diabetes medications, and some may increase kidney problems, especially in older patients; it is important to consult a healthcare provider before taking one. From a general point of view, these substances might be well tolerated in older patients, but caution should be exerted in their use until their efficacy in pain treatment will be proven with appropriate controlled clinical trials, indicating that they cannot be dealt with the same way as drugs with established efficacy.

## 5 VITAMIN B_12_

Among its many functions, vitamin B_12_ (methylcobalamin) is influential in nerve function and red blood cell production. Neuropathy and other neurological issues related to the nervous system can be caused by inadequate vitamin B_12_ intake. Furthermore, some drugs frequently used by diabetic older patients like metformin, proton pump inhibitors (PPI), and histamine (H-2) blockers can all cause a deficiency in Vitamin B_12_ levels [56, 57].

It seems that in patients with DN, vitamin B_12_ deficiency is highly prevalent [58]. In this regard, the prevalence of vitamin B_12_ deficiency in patients with chronic metformin use and the prevalence of DN in patients with normal and low vitamin B_12_ levels was assessed in a cross-sectional study [58]. It was found that DN and vitamin B_12_ levels are inversely related. Also, a lower vitamin B_12_ level was associated with higher metformin doses and male gender [58]. Recently in a randomized, double-blind, placebo-controlled trial, oral treatment with B_12_ 1000 µg/day for one year was studied on DN patients with a mean age of 63. All patients had B_12_ levels less than 400 pmol/L. The results indicated that B_12_ supplementation improved all neurophysiological parameters, sudomotor function, and pain score. Notably, it has been suggested that, especially in diabetic patients aged over 60 years, the lower cut-off values for B_12_ levels, potentially leading to neurological dysfunction if not attained, should be shifted from 150 to 400 pmol/L [56].

It is unclear if vitamin B-_12_ supplements help treat DN, as they are taken along with the usual drug therapy. Including anti-glycaemic therapy aiming at strict glycaemic control, vitamin B_12_ supplements have been the most commonly used supplements [56], all of which are generally considered safe and may offer benefits alongside other pharmacological treatments for older patients with DN. On the other hand, from the quoted studies it appears that the condition in which Vitamin B_12_ may be useful is in the case of vitamin deficiency, a condition that may occur in older patients who are often malnourished and deficient in vitamins. Vitamin supplementation in this case will correct the deficiency, but it is not established whether it may improve neuropathic pain.

## 6 VITAMIN D

In addition to its influence on calcium metabolism, vitamin D has a variety of effects. Deficit of vitamin D is associated with tumors, cardiovascular and autoimmune diseases, and may play a role in diabetes as well as neurodegenerative diseases [59]. Moreover, a growing body of research suggests that vitamin D deficiency may contribute to diabetic sensory motor neuropathy, particularly painful DN [59, 60]. In particular, a neuroprotective effect of vitamin D has been experimentally demonstrated. Vitamin D stimulates the production of nerve growth factor (NGF) [61]. NGF functions through many intracellular signaling pathways and regulates neuronal function, differentiation, growth, survival, and death [62]. Although NGF induces allodynia and hyperalgesia in animals [63], investigators have reported positive results using NGF in human clinical trials of peripheral neuropathic pain [64]. In rats with NGF deficiency, vitamin D treatment has been associated with increased NGF production, which appears to prevent neurotrophic deficits [65]. Further research is necessary to evaluate this phenomenon, but it seems vitamin D through NGF can have a direct impact on nerve function and reduce NP [61, 66]. Besides upregulating NGF production, various studies have demonstrated that vitamin D can act on the nervous system cells by stimulating the production of several neurotrophins, including neurotrophin 3 (NT3) and glial cell line-derived neurotrophic factor (GDNF) [67-71], while downregulating neurotrophin 4 (NT4) [55-59]. In several cases, the stimulation of neurotrophin production by vitamin D was correlated with a neuroprotective effect [65]. In this regard, it is noteworthy that vitamin D has recently been shown to attenuate the hypokinesia and neurotoxicity induced by 6-hydroxydopamine in rats [72]. Within this context, the effect of vitamin D seems to rely on its activity on nerve terminal integrity rather than on direct effects on pain pathways [73]. Accordingly, also the role of NGF remains ambiguous as its neurotrophic activities and involvement in pain-related signalling pathways may play a dual and opposite role in situations such as those related to neuropathy-associated pain [74].

To the best of our knowledge, there are no clinical studies in which the effect of vitamin D administration on DN in the elderly has been evaluated. In light of several clinical and observational studies [75-78], we can conclude that vitamin D use in the general population who are deficient in vitamin D levels might alleviate the symptoms of DN (Many of the participants in the aforementioned studies are around 56-61 years old on average). Given that many older DN patients have already suffered from vitamin D deficiency or insufficiency [79], thus supplementing with vitamin D daily can be an effective way to improve not only DN symptoms but also the predisposing conditions playing an ancillary role in diabetic peripheral neuropathy control [80]. Once again, the main action may rely on vitamin supplementation correcting a deficiency.

## 7 ACETYL-L-CARNITINE

Acetyl-L-carnitine (ALC) is a chemical compound naturally produced in the kidneys and liver and helps to reduce oxidative stress throughout the body. It is believed to be involved in nerve cell function and regeneration [81, 82]. ALC revealed a neuroprotective function in both *in vitro* [83, 84] and *in vivo* studies [85, 86] including antiapoptotic effects in peripheral mononeuropathy models [87].

ALC has also been shown to reduce pain and improve other sensory problems and nerve function in patients with DN, according to several clinical studies [88, 89]. Indeed, treatment efficacy is greater when started early in the disease's course, although more studies are needed to confirm this treatment.

Sima and coworkers reported that ALC (500-1000 mg) administration thrice a day for 1 year in patients with DN, besides improvements in glycemic parameters, was beneficial in pain reduction. Pain relief was reported in terms of improvements in clinical symptom scores and morphometric parameters of sural nerve biopsy (*i.e*. the increased fiber numbers and clusters of regenerating fibers) [89]. An additional clinical study demonstrated that intramuscular injection of ALC followed by oral administration significantly enhanced nerve conduction parameters in DN patients [88]. Furthermore, ALC's effect compared to methylcobalamin in DN patients was assessed in a large multi-centre clinical trial. There was no meaningful difference between the two groups in terms of neuropathy symptom scores and neuropathy disability scores. As a result of both treatments, neurophysiological parameters also improved [90]. A recent Cochrane systematic review focused on four studies (907 participants) examining ALC in DN at doses ranging from 2000 mg/day to 15000 mg/day [91]. The analysis concluded that there is low-quality evidence that ALC reduces pain in DN when compared to placebo after 6 and 12 months. In addition, very low-quality evidence was available on sensations, symptoms, and safety profiles. While, on one hand, ALC therapy may delay the incidence and severity of degenerative disorders of the elderly in prefrail subjects with improved memory and cognitive functions [92], on the other hand, no studies have been conducted to assess the effects of ALC therapy on DN in geriatric population, therefore, high-quality research is needed to determine whether ALC can improve DN associated pain specifically in the elderly.

## 8 ALPHA-LIPOIC ACID

Alpha-lipoic acid (ALA, also known as thioctic acid) is a naturally occurring compound that is also endogenously synthesized by the human body in small amounts. Antioxidants such as ALA could theoretically be effective in treating diabetic peripheral neuropathy [93]. The effectiveness of ALA in reducing symptoms of peripheral DN was evaluated in a meta-analysis of four randomized clinical trials. Intravenous (IV) ALA (600 mg per day for three weeks) significantly reduced the symptoms of neuropathy, whereas oral ALA (600 mg or more per day for three to five weeks) did not significantly reduce neuropathy symptoms. Oral therapy did not seem to be as effective as IV therapy in those studies [94].

The results of another meta-analysis with the participants’ mean age of 57 years showed that treatment with ALA (300-600  mg/day IV for 2-4 weeks) can significantly improve both nerve conduction velocity and positive neuropathic symptoms. Due to the poor methodological quality of most of the studies in this meta-analysis, the evidence may be weak [95]. Moreover, a recent clinical trial on DN patients showed that ALA administration for 3 months ameliorated peripheral DN symptoms and improved metabolic parameters. These results encourage further study of this medication as a possible ancillary treatment for diabetes and subsequently DN [96]. In a recent study, patients with DN (mean age 65) received 600 mg/day orally of ALA for 40 days. Treatment with ALA effectively controlled neuropathy symptoms, fasting triglycerides, and quality of life [97]. However, further clinical trials with stratified age groups are needed to assess what impact ALA has on older patients with DN.

## 9 CAPSAICIN

An important discovery in the field of pain is the identification of the cellular target of capsaicin, called TRPV1. TRPV1 is responsible not only for the hot sensation caused by chili peppers but also for the transduction of noxious stimuli (such as heat) into a painful signal [98, 99]. Furthermore, FDA and the European medicine agency (EMA) have approved the high-concentration capsaicin patch for treating NP in nondiabetic adults. The exclusion of DN-associated pain was only due to the lack of sufficient data in diabetic patients [100]. Nevertheless, capsaicin can be clinically significant in patients suffering from painful DN. In the last thirty years, the application of topical capsaicin cream as an effective agent for localized pain has been increasingly used. It can be summarized that capsaicin cream 0.075%, when applied to painful areas for approximately 8 weeks, is well tolerated and effective in reducing pain, measured by clinical improvements in pain status, walking, working, sleeping, and participation in recreational activities in patients with DN [101-103]. In addition, increasing the dose to 8% of topical capsaicin is equally effective for pain relief in patients with DN [104]. In terms of treatment duration, besides 8 weeks, a few studies indicated the practical use for 3 months [105], or more (1 year) [106, 107]. It should be mentioned that as well as relieving pain-linked foot ulcers, capsaicin can improve glycaemic control and skin microcirculation [108]. The emergence of literature indicating capsaicin administration in patients with type 2 diabetes could improve postprandial hyperglycemia and hyperinsulinemia, suggesting that dietary sources rich in this bioactive compound, like chili peppers, may have a significant impact on diabetic complications [109]. However, clinical trials are required to confirm this hypothesis, especially the combination of capsaicin with other effective pain relief agents in patients with DN.

The mechanism by which capsaicin works involves TRPV1 receptors also denominated vanilloid receptor 1 (VR1) [110]. The discovery from Caterina and co-workers in 1997 on VR1 has changed our understanding of pain mechanisms since it demonstrates that a receptor-coupled channel expressed by nociceptors detects environment stimuli resulting in nociceptor depolarization and consequently produces pain. After that, *in vivo* evidence demonstrated that mice lacking TRPV1 receptors exhibit reduced thermal noxious response and capsaicin-induced paw licking. The whole patch-clamp technique demonstrated that mice lacking TRPV1 receptors present impaired calcium influx in DRG neurons [111]. Therefore, the discovery of TRPV1 was essential to validate capsaicin-induced pain models, which can now be used to study neuronal mechanisms of pain, in addition to testing new TRPV1 antagonists and new drugs that target the consequences of TRPV1 activation in preclinical studies [98, 112].

The mechanism by which capsaicin works is that it causes degeneration or dysfunction of the nerve endings that transmit pain. This way, the nerves become less able to transmit the pain signal. Pain relief is temporary, typically a few months, but capsaicin can be applied again when pain reoccurs. It is commonly believed that the recurrence of pain is due to the regeneration of the nerves. Once the nerves regrow, they will again be able to transmit pain signals to the spinal cord and the brain. However, it has been shown that pain relief persists even after the nerves regain their ability to transmit the pain. This challenges the belief that pain relief caused by capsaicin is related primarily to nerve degeneration or dysfunction [113, 114]. In this intriguing contest, capsaicin might have clinically significant effects on older patients suffering from painful DN.

## 10 PALMITOYLETHANOLAMIDE

N-Palmitoylethanolamide (PEA), a non-endocannabinoid lipid mediator, is of particular interest among the disease-modifying molecules [115]. PEA is able to promote the resolution processes of neuroinflammation and pain [115-117]. In several preclinical studies, PEA was found to reduce inflammation and pain induced by acute stimuli [118-120]. Moreover, in a neuropathic pain model, PEA reduced microglial activation in the spinal cord and increased IL-10 production, an anti-inflammatory cytokine [115]. PEA was found to reduce chronic and neuropathic pain associated with several pathological conditions, including those caused by central neuroinflammation [115]. It is suggested that PEA does not induce tolerance and its efficacy increases progressively with treatment time [121, 122]. Notably, an open-label clinical trial conducted on diabetic patients between the ages 53-86 who suffered from DN found highly significant reductions in pain severity and related symptoms (both *p* < 0.0001) after 600 mg daily administration of PEA for 60 days [123]. Interestingly, using PEA in combination with standard treatments for NP (such as carbamazepine, pregabalin, and oxycodone), pain-relieving effects were observed suggesting a synergic effect of the combined treatment [124]. It is likely that the additive or synergistic effect results from the different mechanisms of action of classical therapies, which act primarily on neurons, as opposed to PEA, which acts mainly on non-neuronal cells, like mast cells and microglia. Once again, it should be emphasized that the clinical usefulness and role of PEA in NP must be further studied and confirmed in well-designed, randomized, age-stratified clinical trials.

## CONCLUSION

When considering substances that have been largely marketed as dietary supplements, a general question arises about the mechanisms involved, whether these mechanisms are still operative in the aged organism, and whether we should consider them as “drug-like mechanisms” at doses that will be substantially higher and administration routes that will be different from those used as dietary supplements or whether we may use them exactly as dietary supplements (for a debate on this point see also Marchesi, Govoni, and Allegri 2022 [81]). The general principles of this discussion apply not only to the selected compounds that have been here discussed in relation to diabetic neuropathy but more in general to the various compounds active under the general umbrella of substances claiming either direct or indirect benefits whenever pain is present. A peculiar aspect in the case of diabetic neuropathy is represented by the fuzzy border between activities acting upon nerve integrity and those working on pain signalling pathways.

Furthermore, the elderly face significant challenges when it comes to NP. Despite its widespread prevalence, DN is commonly undertreated partly due to the age and pathology-associated declining of several physical and mental functions and to the limited treatment options. It is increasingly clear that resources should be refocused and actions taken quickly to produce a paradigm shift in the drug discovery process, need to identify valuable pain relievers purposely for the elderly. This point is supported by the expanding global demographics of the elderly, the evolving value system that guides the approval and reimbursement of new medicines, and the existing gaps in the management of later-life pain, in part summarized in the paper. The latter notion, on one hand, and the limited clinical studies assessing the effectiveness and adverse effects of existing pain management options in this age segment of the population claim the attention of healthcare providers, researchers, and health authorities.

In the end, we strongly believe that dietary supplements should be used in clinical practice and investigated in future clinical trials not as “simple adjuvants” but as mechanism-based treatments to increase the effectiveness of usual treatments implementing their activity or activating other analgesic pathways that are now quite well described.

## Figures and Tables

**Table 1:** Pharmacological management of diabetic neuropathic pain in older patients including one example of comparison between young and old patients.

**Drug**	**Dosage/Duration**	**Outcome**	**Type of Study and ** **References**
Pregabalin	Different doses from 150-600 mg. Without or up to 2 weeks titration period followed by 5 to 13 weeks of treatment.	Significant dose-dependent reductions (> 30%) in endpoint mean pain score on DPRS were observed for pregabalin dosages of 150, 300, and 600 mg/day *versus* placebo for patients aged 65-74 and over 75 years old (in DN* or PHN* patients).	Pooled analysis of 11 clinical studies [38]
Gabapentin *vs.* Amitriptyline	Gabapentin was titrated from 1200 mg/day to a maximum of 2400 mg/day. Amitriptyline was titrated from 30 mg/day to a maximum of 90 mg/day. Both drugs were titrated over 4 weeks and maintained at the maximum tolerated dose for 8 weeks.	All the participants were between the range of 60-83 years old. The outcome was measured by the intensity of pain and paresthesia on categorical scales weekly. Gabapentin produced greater pain reductions than amitriptyline (mean final scores were 1.9 *vs*. 1.3 points below baseline scores; *P* = 0.026). Decreases in paresthesia scores also were in favor of gabapentin (1.8 *vs*. 0.9 points; *P* = 0.004).	An open-label pilot study [39]
Amitriptyline *vs.* Duloxetine *vs.* Pregabalin	Subjects were titrated through 14 days of lower-dose medication (amitriptyline 25 mg twice daily; duloxetine 60 mg every morning; pregabalin 150 mg twice daily) to 14 days of higher-dose medication (amitriptyline 25 mg every morning; 50 mg every night; duloxetine 60 mg twice daily; pregabalin 300 mg twice daily).	The mean age of all the participants were 65 years old. The three study medications all reduced subjective pain with no one drug being superior to another over the 4-week period. (Subjective pain ratings = BPI* severity) showed ∼50% improvement). In terms of adverse effect, Pregabalin promoted sleep, whereas duloxetine increased sleep fragmentation and substantially reduced REM* sleep.	Double-blind, randomized clinical study [40]
Controlled-release Oxycodone	Patients underwent 10 mg of oxycodone every 12 hours. The dose was increased, approximately weekly, to a maximum of 40 mg every 12 hours for 4 weeks.	The mean age of the patients was 63 years old. oxycodone resulted in significantly lower mean daily pain, steady pain, brief pain, skin pain, and total pain and disability in compared to placebo.	Randomized, controlled clinical study [41]
Duloxetine	Patients in 2 age groups (≥ 65 and < 65 years) were stratified by age and then were randomized to duloxetine 60 mg once-daily, 60 mg twice-daily, or placebo for 12 weeks, followed by a 52-week extension phase (re-randomization to routine care or duloxetine 120 mg/day).	The estimated probability of a 30% reduction in pain at endpoint for the older subgroup was 73% for duloxetine 60 mg, and 72% for duloxetine 120 mg (*p* < 0.001 for both duloxetine groups *vs.* placebo). The probability of a 50% pain reduction at endpoint for the older group was 55% for duloxetine 60 mg, and 49% for duloxetine 120 mg (*p* < 0.01 for both duloxetine groups *versus* placebo). The probabilities of response for the younger subgroup were similar to those seen in older patients. There were no significant differences between age groups in BPI Interference average scores at endpoint, and both older and younger patients improved significantly on duloxetine 60 and 120 mg *versus* placebo at endpoint (*p* < 0.05).	Post-hoc analysis of the efficacy and safety of duloxetine from 3 pooled double-blind, placebo-controlled trials [42]

**Abbreviations:** *BPI = Brief Pain Inventory, DN = Diabetic neuropathy, PHN = Postherpetic neuralgia, REM = Rapid eye movement.

## LIST OF ABBREVIATIONS

ADA: American Diabetes Association
ALA: Alpha-lipoic Acid
ALC: Acetyl-L-carnitine
DN: Diabetic Neuropathy
IV: Intravenous
NGF: Nerve Growth Factor
NP: Neuropathic Pain
PEA: Palmitoylethanolamide
SNRIs: Serotonin and Norepinephrine Reuptake Inhibitors
VR1: Vanilloid Receptor 1
